# Comparing decision-support systems in adopting sustainable intensification criteria

**DOI:** 10.3389/fgene.2015.00023

**Published:** 2015-02-11

**Authors:** Bouda Vosough Ahmadi, Dominic Moran, Andrew P. Barnes, Philippe V. Baret

**Affiliations:** ^1^Land Economy, Environment and Society Research Group, Scotland’s Rural College, Edinburgh, UK; ^2^Agronomy, Agroecology, Earth and Life Institute, Université catholique de Louvain, Louvain-la-Neuve, Belgium

**Keywords:** decision-support systems (DSS), sustainable intensification, farm animal genetic resources, models, social science

## Abstract

Sustainable intensification (SI) is a multifaceted concept incorporating the ambition to increase or maintain the current level of agricultural yields while reduce negative ecological and environmental impacts. Decision-support systems (DSS) that use integrated analytical methods are often used to support decision making processes in agriculture. However, DSS often consist of set of values, objectives, and assumptions that may be inconsistent or in conflict with merits and objectives of SI. These potential conflicts will have consequences for adoption and up-take of agricultural research, technologies and related policies and regulations such as genetic technology in pursuit of SI. This perspective paper aimed at comparing a number of frequently used socio-economic DSS with respect to their capacity in incorporating various dimensions of SI, and discussing their application to analyzing farm animal genetic resources (FAnGR) policies. The case of FAnGR policies was chosen because of its great potential in delivering merits of SI. It was concluded that flexible DSS, with great integration capacity with various natural and social sciences, are needed to provide guidance on feasibility, practicality, and policy implementation for SI.

## INTRODUCTION

The growing human population, and growing global demand for food, are major challenges that will need to be addressed in a world with a potentially dramatically changing climate, and with diminishing natural resources such as farm animal genetic resources (FAnGR; Tilman et al., 2011; Tscharntke et al., 2012). These challenges may require a re-appraisal of the capacity to increase food production, especially livestock products without damaging the important environment and ecosystem services they provide. This is referred to as sustainable intensification (SI) approach by some researchers and politicians (Pretty et al., 2011; Tilman et al., 2011; Garnett et al., 2013) but contested by others (Reed, 2012; Kuyper and Struik, 2014; Loos et al., 2014). SI is a multifaceted concept incorporating the ambition to increase or maintain the current level of agricultural yields while reducing negative ecological and environmental impacts by using a broad range of production methods and technologies and by altering consumption patterns. Four key criteria of SI listed by Godfray and Garnett (2014) include: (i) increase or maintain yield, (ii) reduce or maintain land use, (iii) reduce negative environmental and ecological externalities, and (iv) consider/use all forms of agriculture without prejudice. To achieve the objectives of SI by implementing these four main criteria, agricultural, ecological and environmental policies and regulations need to be adjusted accordingly. Policy decisions are often supported and informed by the results of scientific and socio-economic research. Decision-support systems (DSS) are considered as set of scientific and analytical tools and approaches that are used in interpreting research results into policy relevant outcomes.

Decision-support systems can be used to assist agricultural systems’ players and policy makers to achieve objectives of SI by incorporating these four criteria and their subsets in such analytical frameworks. DSS are often used at farm level in informing farmers to improve plans and decisions. They are also used to assist policy makers to evaluate and *ex ante* assessment of future policies. Results of DSS provide an optimum plan of action that can be applied to enterprise, farm, regional, national, or global levels (Geels and Schot, 2007). At farm level, in addition to bio-physical variations of farms (i.e., technical characteristics), goals, and perception of farmers about their farming and agri-ecological systems, as well as their risk attitudes (i.e., social characteristics) vary considerably. Traditionally bio-physical and technical characteristics including technical coefficients, representing specific production functions, are included in certain DSS in form of constraints and activity requirements. However, inclusions of social characteristics and agri-ecological/environmental externalities of farming practices, farmers’ perceptions, behaviors and attitudes in these frameworks are proved to be challenging and less comprehensive (Vanwindekens et al., 2013).

Another challenge in developing and using DSS relevant to policy analysis is inclusion of public and private goods characteristics. Agriculture is inherently multifunctional and often includes both private and public good such as producing food, fiber, etc., with having a profound impact on economies, ecosystems, and environment (Pretty et al., 2001). Farming practices are considered as business activities that generate products and income for farmers (private good) but at the same time could generate positive (e.g., ecosystem services) and negative externalities affecting environmental and ecological systems (public good). Estimating financial performance of farming practices is relatively easy and is routinely done at farm or sector levels using budgeting techniques. However, incorporating agri-ecological/environmental costs and benefits of farming practices (i.e., public good element) is challenging and require getting support from other methodologies. For example the total economic value (TEV) approach is often used to capture these costs and benefits. Direct use value, indirect use value, option value, bequest value, and existence value are components of TEV (Pearce and Moran, 1994). Some of these components also provide a mixture of private and public goods. Other approaches that could support and inform DSS in assessing agri-ecological costs and benefits are: empirical approaches, willingness to pay, contingent valuation, hedonic pricing, and use of experimental data (Randall, 2002). The capability and capacity of DSS to adopt these approaches and capture public/private values vary substantially and therefore both developer and end-user of the results of DSS need to be aware of these differences. In addition, reducing negative ecological and environmental externalities is an important criterion of SI. To go beyond this, “positive” externalities such as ecosystem services could be integrated in DSS.

To incorporate SI’s criteria including social, ecological, and environmental externalities in DSS that ultimately enhance agricultural policies, greater integration of social and technical aspects of farming practices is needed. A wide range of DSS have been developed and applied to different production and agricultural system (Janssen and van Ittersum, 2007). The objectives of this paper are to revisit and compare the capacity of six widely used DSS in adopting the four key SI criteria, agro-ecological/environmental externalities and socio-technical aspects of farming practices, and to discuss the application of DSS to analyzing policies related to conservation of FAnGR.

## REVIEW OF DSS

Agricultural systems and practices are studied using both sociological (anthropological) science methods and technical/engineering sciences. DSS applied to agricultural systems often use statistical and mathematical modeling techniques and are classified based on their purpose, methodology, and assumptions. On this basis, DSS are classified under four main categories: empirical, mechanistic, positive, and normative (Hazel and Norton, 1986). Empirical models are built using observed data and aiming to discover relationships that were not expected *ex ante*. Mechanistic models are built on existing scientific theory and knowledge and are mainly used for *ex ante* scenario analysis (Janssen and van Ittersum, 2007). DSS could be developed using either positive or normative approach. Positive approach tries to mimic the actual behavior of the farmers or managers whereas normative approach tries to find optimum solution for a given system.

### COMPARISONS

Six DSS approaches namely: structural equation modeling (SEM), linear- and non-linear programming (LP and NLP), positive mathematical programming (PMP), multiple criteria decision making (MCDM), cognitive mapping (CM), and dynamic programming (DP) were selected for comparison in this study. Table 1 summarizes the characteristics of the mentioned DSS approaches. Among the selected DSS approaches, SEM and CM are considered as empirical methods that are mainly used in *ex post* analysis aiming at revealing relationships in observed data that will be used to predict outcomes in future. Technical aspects of farming practices could be included to some extend in these methods but less than mechanistic models. Both SEM and CM are strong in looking at social aspects of socio-ecological farming systems including farmers, behavior, perceptions, and goals. These social attributes could be related to ecological and environmental issues and therefore could provide useful insight. Considering mentioned characteristics the potential of these methods in assisting with SI merits are judged to be moderate to high.

**Table 1:** **Comparisons of selected DSS including characteristics and their capacity of integrating SI criteria and socioeconomic aspects**.

**Method**	**Descriptive**						Capacity of integrating*						
							Sustainable intensification criteria**				**Positive externalities (e.g., ecosystem services)**	**Social aspects (goals)**	**Attitude (e.g., risk)**
	**Type**	**Level**	**Time aspect**	**Model inputs**	**Objective and assumptions**	**Technical**	**S1**	**S2**	**S3**	**S4**			
SEM	Empirical	Farm	Retrospect (*ex post*)	Independent and explanatory variables	Explore relationships in observed data	No	Yes	No	Yes	Yes	Yes (M)	Yes	Yes
LP and NLP	Mechanical/normative or positive	Animal/crop/farm	Season/year/static/dynamic/(*ex ante*)	Activities, constraints, coefficients	Maximizing or minimizing objectives Linear/non-linear relationships	Yes	Yes (L)	Yes	Yes	Yes	Yes (VH)	Ye(L)	Ye(L)
PMP	Mechanical/positive	Animal/crop/farm	Season/year/static/dynamic/(*ex ante*)	Activities, constraints, coefficients	Mimic actual farm practice. Linear/non-inear relationships	Yes (L)	Yes (L)	Yes	Yes	Yes	Yes (H)	No	Yes (L)
MCDM	Mechanica	Farm/region/country	Season/year/static/dynamic/(*ex ante*)	Goals as objectives, constraints	One goal optimized, constraints on others get tighter	Yes (L)	Yes	Yes	Yes	Yes	Yes (H)	Yes (H)	Yes (H)
CM	Empirical	Individual (farm)/socia	Year (*ex post*)	Quantitative, operations, drivers, constraints	ncorporate social aspect in DSS, farmer	Yes (L)	Yes	Yes (L)	Yes (L)	Yes (L)	Yes (VH)	Ye (VH)	Yes (VH)
DP	Mechanical/normative	Animal/crop/farm	Year/dynamic (*ex ante*)	Pay-off values, transition probabilities	Profit maximizing	Yes (L)	No	Yes	Yes (VL)	Yes (VL)	Yes (M)	No	Yes (L)

“Capacity of integrating was specified by “yes” or “no” The foiiowing scale was used for the “yes” category: VL, very limited; L, limited; M, moderate; H, high; VH, very high. **S1, increasing/maintaining yield; S2, using less land or maintaining current land usage; S3, less environmental and ecological damage, more biodiversity and ecosystem services; S4, utilizing all types of technology without prejudice. SEM, structural equation modeling; LP, linear programming; NLP non-linear programming; PMP positive mathematical programming; MCDM, multiple criteria decision making; CM, cognitive mapping; DP dynamic programming.

LP/NLP, PMP, MCDM, and DP are considered as mechanical approaches that are built based on theory and knowledge to find solutions for management problems in relation to farming systems by running the models under different scenarios and policy assumptions. LP/NLP and PMP have good capacity in incorporating technical aspects of the systems (including economics, production, environment, and ecology/biodiversity; Cypris, 2000; Vosough Ahmadi et al., 2011, 2015; Stott et al., 2012). However, they have fairly limited capacity in covering social and behavioral characteristics of the farmers. MCDM approaches are less sophisticated in terms of the level of technical details of the systems but could potentially include different goals of various stakeholders or certain view points (i.e., goals). Social and technical aspects of environmental and ecological issues could be covered to some extend by these approaches. DP is an example of DSS approaches that could assist farm managers with decisions within a short time (Kennedy, 1986). They are capable to incorporate risk and stochastic events but relatively low complexity of the system could be built in these models (Stott, 1994). They are not usually capable of inclusion of social aspects and not very strong in incorporating environmental and biodiversity elements. In terms of capability of inclusion of SI goals and merits, in our judgment LP/NLP and CM are considered as approaches with a very high capacity. After these methods, MCDM are considered as highly capable in incorporating and helping with SI concept. SEM and DP are considered as moderate in terms of their capability of adopting SI criteria.

## INTEGRATING SI CRITERIA IN DSS

In the following lines the application and usefulness of the mentioned DSS approaches in relation to SI’s four criteria definition by Godfray and Garnett (2014), is discussed.

*(S1) Increasing/maintaining yield (intensification aspect)*: This criterion is related to utilizing technologies and also to some extent to improving management of crops and livestock (e.g., controlling diseases) that leads to higher yield. DSS could help with informing decision makers with control of diseases, and short and long term optimum management for example in relation to keeping/replacement of animals or crop rotation etc.

*(S2) Using less land or maintaining current land usage (intensification aspect)*: DSS and in particular mechanistic models could provide insight on the impact of reducing available land on production and could suggest alternative solutions if technology allows.

*(S3) Less environmental and ecological damage, more biodiversity and ecosystem services (sustainability aspect)*: This condition could potentially be included at both technical and social levels in DSS models. However, in majority of the available models environmental and ecological aspects have been added as constraints to the systems whereas it could be considered as objective of the farming in these models.

*(S4) Utilizing all types of technology without prejudice (both intensification and sustainability elements)*: There is an on-going debate about this criterion of SI (Loos et al., 2014). In DSS approaches such as CM and MCDM, the perceptions and goals of farmers with respect to using particular technologies to improve/increase yield or to protect environment and biodiversity could be analyzed and included in the models. In this case individual and social believes/perceptions of farmers that are added to model will assist policy makers to come up with effective policies.

All the four mentioned SI criteria could be considered as objectives and opportunity or could be as constraints of the agricultural systems in DSS models. Similarly they are influenced by short and long term goals, and perceptions and behavior of farmers. These criteria are also directly related to technological advances that help with increase/maintain yield but lowering negative externalities and also by increasing efficiency.

## APPLICATION TO FAnGR POLICIES

In the context of FAnGR conservation and biodiversity policies, the issue of allocation of limited preserving genetic diversity budget in determining actual conservation priorities among endangered species has been included in a number of theoretical and operational DSS by a number of authors (Weitzman, 1998; Naidoo and Iwamura, 2007). In most of these DSS, objective was to preserve maximum diversity given the limited financial, technological, and perhaps logistical resources. Probability of extinction has been a core element of conservation DSS modeling. In addition, discounting future benefits and costs as a basis for economic justification of conservation policies has been taken into account. More recent applications of DSS to FAnGR context showed that supply chain management, cooperation, management of common goods in relation to biological resources and data management are important elements that need to be considered in developing and using DSS (Labatut et al., 2010, 2012). A number of other areas for consideration are: the goals of conservation, intrinsic value of breeds, public and private good elements of FAnGR, the impact of genetic technologies on society and power in the breeding systems. Also the impact of demand of agricultural product and services on commodity market prices at farm level that are not usually explicitly included in DSS models must be incorporated in the models subject to data availability.

Means to SI in livestock production or in other words means to improve sustainability and productivity of farm animals need to be sought through breeding, genetic engineering, nutrition, health, and welfare. For example new phenotypes linked to sustainable animal productivity could be developed and integrated into breeding schemes. SI’s merits could also be achieved through economically justified conservation of FAnGR that depends on the increased adaptive capacity in response to change that such preservation in a genome resource bank offers beyond that of alternatives. The technological preservation of FAnGR does not only require economic and scientific input, to direct optimal decision making, but social science methods to reflect the historical, cultural, and social aspects of genetic resources at farm level and beyond. DSS therefore play a crucial role in integrating both technical and social aspects of farming practices and provide an improved policy and practical guidance to tackle major global challenges ahead.

### Conflict of Interest Statement

The authors declare that the research was conducted in the absence of any commercial or financial relationships that could be construed as a potential conflict of interest.
